# Effect of Dietary Digestible Protein Levels on Muscle Growth and Oxidative Stress in Amazonian Pintado (*Pseudoplatystoma reticulatum* × *Leiarius marmoratus*)

**DOI:** 10.3390/biology13100825

**Published:** 2024-10-15

**Authors:** Stephane Vasconcelos Leandro, Daniel Rabello Ituassú, Valéria Dornelles Gindri Sinhorin, Danilo Henrique Aguiar, Paula Sueli Andrade Moreira, Ana Julia Lopes Braga Ferneda, Soraia Andressa Dall’Agnol Marques, Adilson Paulo Sinhorin, Anderson Corassa, Ana Paula Silva Ton, Leonardo Willian de Freitas, Maicon Sbardella

**Affiliations:** 1 Instituto de Ciências Agrárias e Ambientais, Universidade Federal de Mato Grosso, Sinop 78.550-725, MT, Brazil; svl.zootecnista@gmail.com (S.V.L.); paula.moreira@ufmt.br (P.S.A.M.); anderson.corassa@ufmt.br (A.C.); ana.ton@ufmt.br (A.P.S.T.); lwillianf86@gmail.com (L.W.d.F.); 2 Empresa Brasileira de Pesquisa Agropecuária (EMBRAPA Agrossilvipastoril), Sinop 78.550-000, MT, Brazil; daniel.ituassu@embrapa.br; 3 Instituto de Química, Universidade Federal de Mato Grosso, Cuiabá 78.060-900, MT, Brazil; valeria.sinhorin@ufmt.br; 4 Instituto de Ciências Naturais, Humanas e Sociais, Universidade Federal de Mato Grosso, Sinop 78.550-725, MT, Brazil; dha.danilo@gmail.com (D.H.A.); adilson.sinhorin@ufmt.br (A.P.S.); 5 Instituto de Ciências da Saúde, Universidade Federal de Mato Grosso, Sinop 78.550-725, MT, Brazil; ajlbraga@hotmail.com; 6 Faculdade de Medicina Veterinária e Zootecnia, Universidade Federal de Mato Grosso do Sul, Campo Grande 79.070-900, MS, Brazil; soraia.agnol@hotmail.com

**Keywords:** free radicals, muscle hypertrophy, nutrition, pisciculture

## Abstract

**Simple Summary:**

In fish farming, protein is the most expensive macronutrient in the diet. Research on the physiology of fish nutrition has focused on the relationships between nutrient intake and animal development and metabolism. The dynamics of protein metabolism are influenced by the body’s nutrient needs and dietary protein and energy intake. The Amazonian Pintado, a crossbred hybrid of *Pseudoplatystoma reticulatum* × *Leiarius marmoratus*, is farmed due to its growth rate, feed conversion efficiency, carcass yield, and absence of intramuscular bones. This study aimed to evaluate the effects of dietary digestible protein levels on muscle growth dynamics and oxidative stress in the white muscle of Amazonian Pintado. The results revealed that high dietary levels of digestible protein, despite increasing protein and free amino acid concentrations in the muscle, restrict muscle hypertrophy and increase tissue exposure to oxidative damage in Amazonian Pintado farming.

**Abstract:**

This study aimed to evaluate the effects of dietary digestible protein levels on the growth dynamics and oxidative stress status of white muscle fibers in Amazonian Pintado (*Pseudoplatystoma reticulatum* × *Leiarius marmoratus*). Four hundred and fifty-five juveniles of Amazonian Pintado were fed diets containing varying digestible protein levels (225, 250, 275, 300, 325, 350, or 375 g kg^−1^) for 75 days. At the end of the experiment, the fish were fasted for 24 h, anesthetized, and euthanized to obtain muscle samples. The linear and quadratic effects of dietary digestible protein levels on white muscle fiber diameter, metabolite concentrations, and oxidative stress were assessed. The results revealed that increasing dietary digestible protein levels linearly raised the concentrations of free amino acids and total proteins in muscle tissue but also led to elevated levels of TBARS, indicating increased oxidative stress. Notably, the average area of muscle fibers with a cell area greater than 1133 µm^2^ decreased, reflecting restricted muscle hypertrophy, whereas glycogen and glucose levels also declined. These findings suggest that although high dietary digestible protein enhances protein and free amino acid concentrations in muscle tissue, it may compromise muscle hypertrophy and increase oxidative damage in Amazonian Pintado, underscoring the complexity of optimizing diet formulation.

## 1. Introduction

Dietary protein is prioritized in fish metabolism for energy production (ATP) due to the scarcity of carbohydrate sources in their natural environment [1,2]. In commercial fish farming, protein constitutes more than 50% of production costs [3]. Thus, formulating diets with lower protein levels and higher carbohydrate concentrations to meet energy demands poses challenges for maintaining fish health, performance, and muscle quality [4].

The dynamics of protein metabolism are influenced by the interplay between the body’s nutritional requirements and dietary intake of protein and energy [5]. Although protein synthesis rates are higher in the liver, muscle tissue plays a significant role in protein metabolism in fish, constituting approximately 60% of body weight [6]. Research on fish nutrition commonly assesses nutritional needs on the basis of zootechnical performance responses [7,8]. However, the metabolic profiles of nutrients in different body compartments impact tissue oxidative status [9]. Therefore, understanding the relationships between diet, oxidative and metabolic status, and muscle development is crucial for enhancing feed formulation and feeding strategies in fish farming systems across different species.

The Amazonian Pintado is a hybrid fish resulting from a cross between female *Leiarius marmoratus* [10] and male *Pseudoplatystoma reticulatum* [11]. In fish farming systems, this hybrid has demonstrated rapid growth, efficient feed conversion, and high carcass yield [12]. Although studies indicate that the Amazonian Pintado exhibits feeding habits and gastrointestinal tract histology consistent with those of omnivorous fish [13], there is currently no evidence demonstrating how diets with omnivorous characteristics impact the metabolic dynamics, oxidative stress, and muscle development of this hybrid. Previous studies have indicated that pure species of the genus *Pseudoplatystoma* require digestible protein levels above 39% for optimal growth [14,15]. Although some investigations have explored different dietary crude protein levels for the Amazonian Pintado [16,17], the specific nutritional requirements for this hybrid remain not well-defined.

The purpose of this study was to evaluate the effects of dietary digestible protein levels on muscle fiber development dynamics, the metabolic profile, and oxidative stress in the white muscle of Amazonian Pintado.

## 2. Materials and Methods

This experiment was conducted at the Fish Nutrition Laboratory of the Brazilian Agricultural Research Corporation (EMBRAPA Agrosilvopastoril), located in the city of Sinop, State of Mato Grosso, Brazil. The experimental procedures involving the use of animals were approved by the Ethics Committee on the Use of Animals of the Embrapa Agrosilvopastoril (protocol no. 002/2020).

### 2.1. Animals, Management, and Experimental Diets

A total of 455 juveniles of Amazonian Pintado (*Pseudoplatystoma reticulatum* × *Leiarius marmoratus*) were used in a completely randomized design with seven treatments, five replicates, and 13 fish per experimental unit. The fish had a mean weight of 299.54 ± 48.03 g and a mean length of 27.69 ± 1.20 cm and were distributed into 500 L tanks. The fish had been previously acclimatized to the facilities for 60 days, followed by a 75-day feeding experiment. The fish were fed twice a day (at 8:00 a.m. and 4:00 p.m.) until apparent satiation.

The fish were fed diets containing increasing levels of digestible protein (225, 250, 275, 300, 325, 350, or 375 g kg^−1^) and a digestible energy of 3400 kcal kg^−1^. The experimental diets were formulated on the basis of fish meal, soy protein concentrate, blood meal, and corn, considering the digestibility values of these ingredients determined for the Amazonian Pintado by Marques et al. [18] (Table 1). The diets were prepared by finely grinding and mixing all the ingredients, followed by extrusion in a twin-screw extruder (Eco Soluções, Viçosa, Brazil) with a 4 mm die, drying in a forced-air oven at 55 °C for 24 h, and storing at 5 °C and 40% humidity.

### 2.2. Collection of Biological Material

At the end of the 75-day feeding trial, the fish were fasted for 24 h. Three fish were randomly selected per tank (15 per treatment), anesthetized with clove oil (50 mg/L) diluted in ethanol (1:20), individually weighed, and euthanized by spinal cord sectioning for the collection of white striated skeletal muscle samples (medial and dorsal regions). The fragments for biochemical analysis were preserved in an ultrafreezer (model IULT486D, Indrel, Londrina, Brazil) at −80 °C, and for histological analysis, they were preserved in 10% buffered formaldehyde.

The fish sampled for the analyses had an average weight of 628.76 ± 18.54 g, an average length of 34.31 ± 0.37 cm, and an average eviscerated carcass weight of 561.30 ± 12.17 g, with similar distributions between treatments.

### 2.3. Muscle Fiber Morphometry

Sections of the muscle fixed in 10% formaldehyde were dehydrated using a graduated series of ethanol (70-to-95%). The sections were subsequently soaked in historesin and sectioned to a thickness of 3 μm using a Leica semiautomatic rotary microtome (model 2245). These sections were rehydrated in distilled water and stained with hematoxylin–eosin (HE).

**Figure 1:**
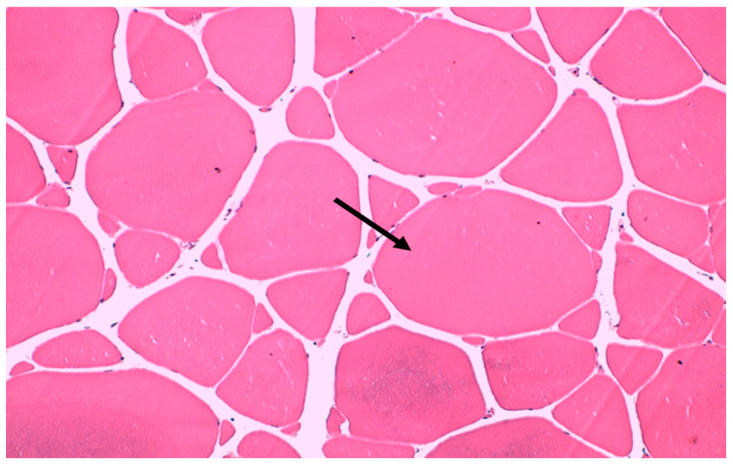
Image of a cross-section of the muscle fibers obtained under a microscope at 20× magnification. The image represents one of the points sampled on a slide. Note the muscle fibers (arrow) used to measure the cell area in μm^2^.

To measure the area and number of muscle fibers, images were taken from five different points on each slide (Figure 1) to determine the number of cells. The area of each muscle fiber in the cross section was measured using Moticam 2.0 software (Motic Asia, Kowloon, Hong Kong).

To evaluate the hyperplastic and hypertrophic growth patterns of the muscles, the muscle fibers were divided into five classes according to the quartiles that included the muscle fiber area, as adapted from Veggetti et al. [19] and Valente et al. [20].

### 2.4. Protein, Free Amino Acid, and Ammonia Contents in Muscle

The protein concentration was determined according to the methods of Bradford [21], using bovine serum albumin as a standard. The results are expressed in mg g^−1^.

The free amino acid content was determined using the Spies [22] method, which involved the addition of 0.5% ninhydrin (diluted in isopropyl alcohol). The absorbance was measured at 570 nm and compared with a standard curve of free amino acids, with the results expressed in mmol g^−1^ tissue.

Ammonia was measured using the method of Gentzkow and Masen [23] via spectrophotometry at a wavelength of 420 nm, and the results were compared to an ammonia standard curve, with the data presented in µmol g^−1^ tissue.

### 2.5. Glycogen, Glucose, Lactate, and Fat Contents in Muscle

The muscle concentrations of the metabolites glycogen, glucose, and lactate were determined via spectrophotometry using the methods described by Bidinotto et al. [24], DuBois et al. [25], and Harrower and Brown [26].

For the measurement of glycogen, the samples were analyzed at a wavelength of 490 nm, and the results were compared to a glycogen standard curve, expressed in µmol g^−1^ tissue.

For glucose measurements, samples were taken at a wavelength of 480 nm using a spectrophotometer (Varian Cary 50 UV-VIS, San Francisco, CA, USA). The results were compared to a glucose standard curve and are expressed as µmol g^−1^ tissue.

For lactate measurement, the samples were read at a wavelength of 570 nm, and the results were compared to a lactate standard curve, presented as µmol g^−1^ tissue.

The fat content of the muscle was determined via Soxhlet extraction following the procedure described by Folch et al. [27]. The results are expressed as mg g^−1^ tissue.

### 2.6. Parameters of Oxidative Damage in Muscle

For the analysis of the biochemical parameters of oxidative stress, the tissues were previously thawed, carefully sampled on an ice-cold surface, and weighed in the proportions recommended for each analysis.

#### 2.6.1. Thiobarbituric Acid-Reactive Substances (TBARSs)

The substances reactive to thiobarbituric acid were measured using the method described by Buege and Aust [28]. The concentration of TBARS was expressed as nmol MDA mg^−1^ protein, following the calibration curve for MDA.

#### 2.6.2. Protein Carbonylation

For the analysis of protein carbonyl groups, the method described by Colombo et al. [29] was used. The results are expressed as nmol Carbonyl mg^−1^ protein.

#### 2.6.3. Antioxidant Complex

The activities of the antioxidant enzymes superoxide dismutase (SOD) and glutathione-S-transferase (GST), as well as the nonenzymatic antioxidant reduced glutathione (GSH) in the muscle, were measured following the methodologies described by Misra and Fridovich [30], Habig et al. [31], and Sedlack and Lindsay [32].

### 2.7. Statistical Analysis

The data were analyzed using the SAS Glimmix procedure (SAS Institute, Inc., Cary, NC, USA), considering the levels of digestible protein in the diet as the main factor and the muscle sample of each fish as the experimental unit. All data were tested for homogeneity of residual variance, normality of residuals, linearity, and outliers. Influential data (outliers) were removed from the analysis when they exceeded two standard deviations from the mean of the residuals, resulting in the exclusion of 2 data points for biochemical parameters and 3 for oxidative stress parameters.

Linear and quadratic polynomial regression analyses were performed to determine the dose–response effects of dietary digestible protein levels on the response variables. For the histological data, the eviscerated fish carcass weight was included in the model as a covariate. Linear or quadratic effects (*p* < 0.10) were plotted.

## 3. Results

### 3.1. Dynamics of Muscle Fiber Development

**Figure 2:**
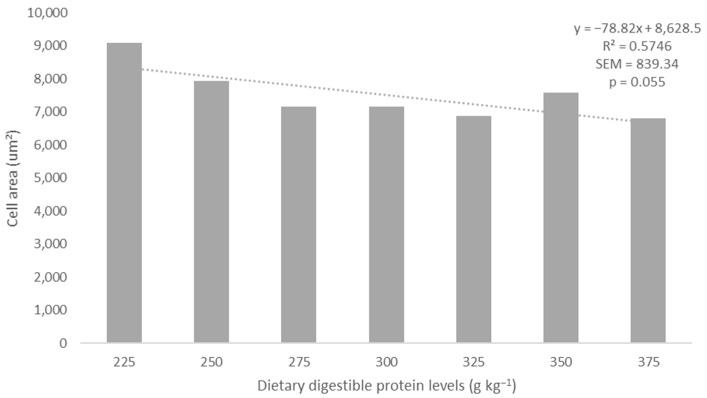
The increase in dietary digestible protein levels linearly reduced (dotted line) the mean sectional cell area of Class 5 muscle fibers (greater than 1133 μm^2^).

Increasing dietary digestible protein levels linearly decreased the average area of muscle fibers with a cell area greater than 1133 µm^2^ (Y = −278.82x + 8628.5; R^2^ = 0.575; *p* = 0.055; Figure 2). No differences were detected (*p* > 0.05) in the number of white fiber muscle cells across different cell area sizes (Table 2).

### 3.2. Proteins, Free Amino Acids, and Ammonia

**Figure 3:**
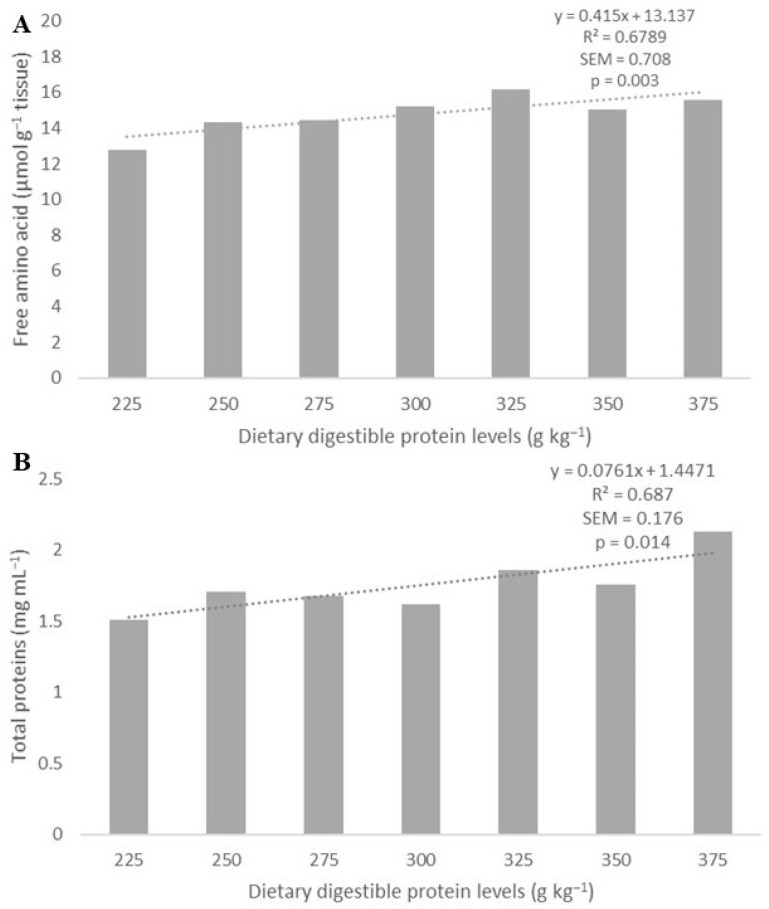
The increase in dietary digestible protein levels linearly increased (dotted line) free amino acid levels (**A**) and total protein concentration (**B**) in the muscle tissue of Amazonian Pintado.

The concentrations of free amino acids (Y = 0.4158x + 13.137; R^2^ = 0.6801; *p* = 0.003; Figure 3A) and total protein (Y = 0.0761x + 1.4471; R^2^ = 0.687; *p* = 0.014; Figure 3B) in the muscle tissue increased linearly with increasing dietary digestible protein levels, whereas the ammonia concentration was not affected (*p* > 0.05) (Table 3).

### 3.3. Muscle Metabolites

**Figure 4:**
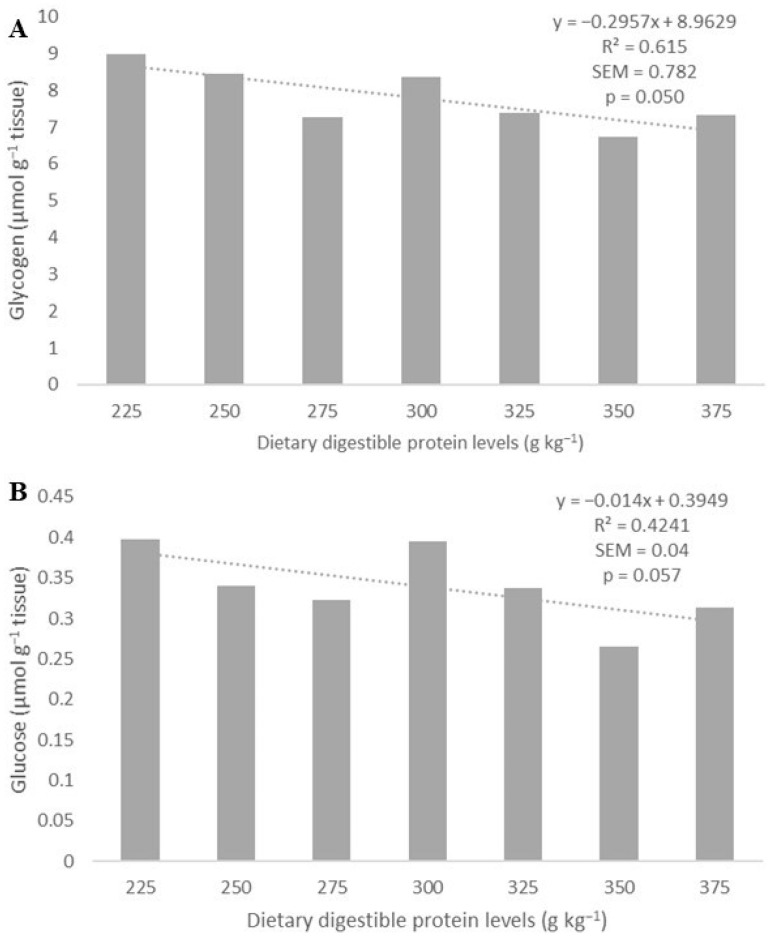
The increase in dietary digestible protein levels linearly reduced (dotted line) glycogen (**A**) and glucose (**B**) levels in the muscle tissue of Amazonian Pintado.

The levels of glycogen (Y = −0.2947x + 8.9597; R^2^ = 0.6134; *p* = 0.0503; Figure 4A) and glucose (Y = −0.014x + 0.3949; R^2^ = 0.4241; *p* = 0.0574; Figure 4B) in the muscle tissue decreased linearly with increasing dietary digestible protein levels, whereas lactate was not affected (*p* > 0.05) (Table 4).

### 3.4. Oxidative Stress in Muscle Tissue

**Figure 5:**
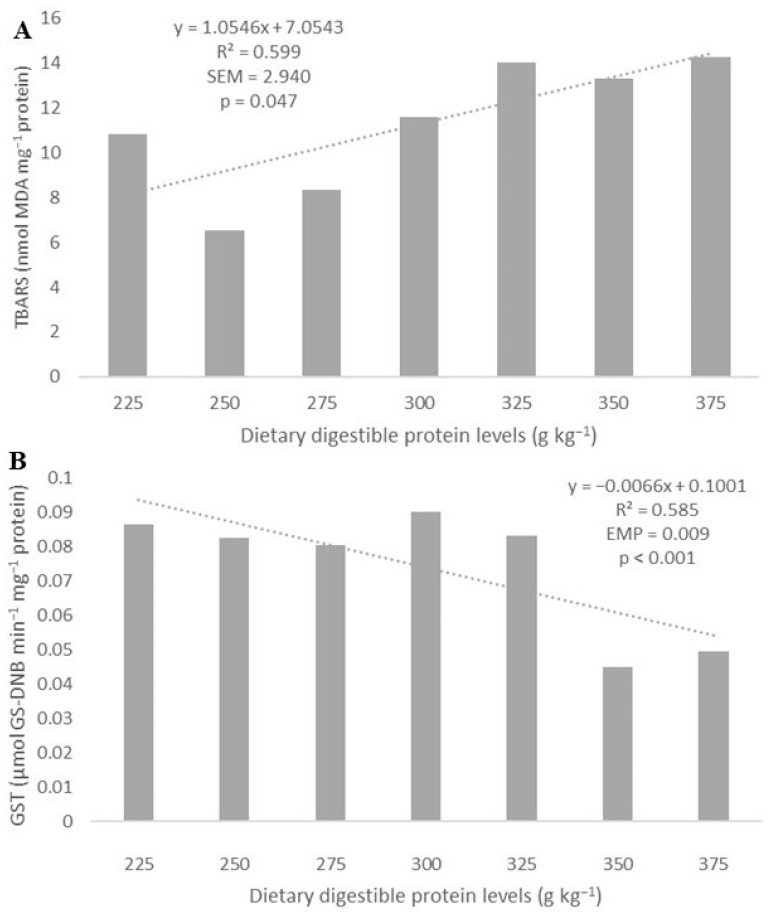
The increase in dietary digestible protein levels linearly increased (dotted line) thiobarbituric acid reactive substances (TBARSs) (**A**) and reduced (dotted line) GST activity (**B**) in the muscle of Amazonian Pintado.

The concentration of TBARS increased linearly with increasing dietary digestible protein levels (Y = 1.0543x + 7.0566; R^2^ = 0.5986; *p* = 0.047; Figure 5A), whereas the concentration of carbonyl proteins was not affected (*p* > 0.05) (Table 5).

The GST activity decreased linearly with increasing dietary digestible protein levels (Y = −0.0066x + 0.1001; R^2^ = 0.5847; *p* = 0.0001; Figure 5B), whereas the SOD and GSH activities were not affected (*p* > 0.05) (Table 6).

## 4. Discussion

Several factors, such as water temperature, salinity, stocking density, duration of confinement, and feeding practices, influence the distribution of myocyte numbers and size during fish growth [33,34]. In this study, increased levels of dietary digestible protein led to a reduction in the mean diameter of muscle cells with an area greater than 1133.73 µm^2^ (Class 5), indicating decreased protein deposition (atrophy) in muscle fibers. Muscle hypertrophy is contingent on the balance between protein synthesis and degradation [35]; a higher protein synthesis rate can lead to increased muscle mass, whereas energy-restricted diets may drive protein degradation to meet metabolic needs [36,37,38]. In this study, diets with higher digestible protein levels contained proportionally lower concentrations of carbohydrate sources, potentially increasing the demand for protein degradation for energy production, which limits muscle protein deposition.

In contrast to the findings of this study, Silva et al. [39] reported that increasing dietary protein content (from 20 to 60%) resulted in increased fiber recruitment and muscle fiber area in *Pagellus bogaraveo*, a carnivorous species. Although the metabolic characteristics of the Amazonian Pintado are not fully understood, its feeding habits and gastrointestinal tract histology suggest similarities with those of omnivorous fish [13]. Therefore, the observation that Amazonian Pintado fed diets with lower levels of digestible protein prioritize dietary amino acids for growth rather than for energy production (protein-sparing effect) supports the hypothesis that the metabolism of these hybrids resembles that of omnivorous fish. This finding indicates greater efficiency in utilizing dietary carbohydrates as an energy source [38].

In this study, increasing dietary digestible protein levels resulted in a reduction in muscle glycogen and glucose levels, alongside an increase in free amino acids and total protein without affecting fat accumulation. This pattern aligns with other studies that reported similar trends in various fish species. Lin et al. [40] reported increased muscle glycogen content with increasing starch levels (5%, 10%, and 20%) in the diet of *Micropterus salmoides*. Kirchner et al. [41] and He et al. [4] reported a direct relationship between the dietary protein content and free amino acid concentration in the muscle tissue of rainbow trout and *Carassius carassius* triploid, respectively. However, Yamamoto et al. [42] and Jiménez et al. [43] reported no relationship between dietary protein levels and free amino acids in the muscle of rainbow trout and *Dentex dentex*, respectively. Therefore, although the concentration of free amino acids in muscle tissue is associated with muscle fiber synthesis [4], energy status appears to determine the direction of metabolic processes (either protein degradation or synthesis).

In diets with protein levels exceeding developmental requirements, excess protein is degraded into free amino acids and converted into energy in the liver, leading to increased visceral fat deposition and ammonia excretion [44]. In fact, this study revealed that although increasing dietary protein levels did not affect growth performance or meat quality, it did increase ammonia excretion and reduce visceral fat and liver weight [45]. Despite the lack of significant effects on growth performance, these findings from our experiment suggest metabolic adjustments to higher dietary protein intake, which merit further investigation. In this context, it was observed that fish receiving lower protein diets presented a better protein efficiency rate, suggesting a potential trade-off between dietary protein concentration and growth dynamics. Moreover, the reduced fat accumulation in carcasses from diets above 325 g kg^−1^ of digestible protein further underscores the complexity of optimizing protein levels to balance growth and body composition in this species. Additionally, the protein profile in the diet plays a central role in the concentration of free amino acids in fish muscle, as observed when fish meal is replaced with vegetable proteins (soy flour, corn gluten meal, wheat gluten, and peanut flour) [46]. Thus, the accumulation of total protein and free amino acids in muscle tissue, along with smaller muscle fiber diameters, may reflect the amino acid profile of dietary protein and substrate availability for energy production in metabolism.

The influence of dietary protein–carbohydrate–fat profiles on oxidative status has been extensively discussed in the literature. For example, Bai et al. [47] investigated the impact of dietary carbohydrate levels on growth and oxidative stress in *Megalobrama amblycephala* × *Culter alburnus* (BT) and *Culter alburnus* (TC) hybrids. Similarly, Álvarez et al. [48] examined the effects of dietary digestible protein levels on rainbow trout (335, 339, 406, or 409 g kg^−1^) and sea bass (277, 342, 378, or 412 g kg^−1^), highlighting the increased susceptibility of muscle tissue to oxidation with high dietary protein levels. Additionally, Jimenez et al. [49] explored the effects of macronutrient proportions on *Dentex dentex* diets and reported that diets with high lipid contents induced greater lipid peroxidation. These findings collectively indicate increased lipid peroxidation in fish fed diets with increased digestible protein and decreased carbohydrate concentrations, which is consistent with the observations in this study.

Oxidative stress refers to damage inflicted on cells and tissues by reactive oxygen species (ROS) when the antioxidant defense system, which is composed of enzymatic and nonenzymatic components, fails to neutralize them [49,50,51]. During lipid peroxidation, ROS are converted into less reactive secondary metabolites through the action of the antioxidant system in the termination phase of lipid peroxidation reactions [52]. The accumulation of thiobarbituric acid reactive substances (TBARSs) serves as an indicator of oxidative damage. In addition to lipid peroxidation, increased ROS cause protein damage through the formation of carbonyl groups, altering the structure and function of amino groups in proteins [53,54]. In our study, although carbonylated protein remained unaffected, there was an increase in muscle TBARS accumulation with increasing dietary digestible protein levels, suggesting heightened susceptibility to oxidative damage.

Cells protect against ROS-induced oxidative damage through antioxidant enzymes, including superoxide dismutase (SOD), catalase (CAT), and glutathione peroxidase (GPx) [54]. SOD acts as a primary defense mechanism against the toxic properties of superoxide radicals [55,56]. Research on *Pelteobagrus fulvidraco* demonstrated that SOD activity increased while glutathione (GSH) activity decreased with increasing dietary protein levels, suggesting that elevated dietary protein could facilitate the synthesis of immunological antioxidant enzymes to maintain the stability of the antioxidant system and ensure animal health [57]. Furthermore, glutathione-S-transferase (GST) plays a crucial role in detoxifying xenobiotics by conjugating reduced glutathione (GSH), with glutathione reductase (GR) replenishing GSH levels to serve as an essential antioxidant and cofactor for GST [55,58]. Our study suggests a potential reduction in GST activity at higher dietary digestible protein levels, with increased stress represented by elevated TBARS levels, given the lack of additional GSH generation and the unchanged SOD activity. This adaptation in protein metabolism may induce greater oxidative stress in the animal, as evidenced by our findings. The experimental diets used in this study were designed with a focus on achieving the desired levels of digestible protein by maintaining constant digestible energy. However, it is important to acknowledge that variations in carbohydrate-to-lipid ratios and ingredient selection could have impacted the metabolic responses observed, as noted by Calzada-Ruiz et al. [59] and Guerrero-Zárate et al. [60]. Although our study successfully met the primary objective of evaluating the effects of digestible protein levels, adjustments in lipid and ingredient proportions may provide further avenues for refining diet formulations and improving nutritional outcomes in future studies.

The findings of this study have significant implications for fish farming practices, particularly in optimizing dietary formulations for Amazonian Pintado. By understanding the relationship between dietary digestible protein levels and muscle growth dynamics, producers can tailor feed compositions to enhance growth rates and feed efficiency. This optimization could lead to reduced feed costs, which are a major component of operational expenses in aquaculture. Furthermore, prioritizing sustainable dietary sources, such as plant-based proteins, may mitigate the environmental impacts associated with fish meal production, contributing to more eco-friendly aquaculture practices. Ultimately, integrating these insights into feeding strategies could improve both the economic viability and sustainability of fish farming operations.

## 5. Conclusions

This study demonstrated that high levels of digestible protein in the diet of Amazonian Pintado led to increased concentrations of muscle protein and free amino acids, restricted the hypertrophy of white fibers, and increased tissue exposure to oxidative damage compared with diets containing lower digestible protein levels. Further research is needed to examine the effects of different protein sources and their long-term impacts on muscle development and oxidative stress, which will enhance our understanding of optimal feeding strategies and sustainable aquaculture practices.

## Figures and Tables

**Table 1:** Composition of the experimental diets (as dry matter).

Ingredients (%)	Digestible Protein (g kg^−1^)						
	225	250	275	300	325	350	375
Corn ^1^	56.09	45.59	37.50	37.16	34.23	13.00	21.25
Fish meal ^2^	20.00	20.00	20.00	30.00	35.00	40.42	42.01
CPC ^3^	12.49	20.48	25.94	24.80	20.17	19.00	21.04
Soybean oil	5.48	8.08	9.54	7.27	5.38	9.59	5.48
Blood meal ^4^	3.00	3.00	5.00	0.00	5.00	10.00	10.00
Dicalcium phosphate	1.83	1.74	1.18	0.11	0.00	0.00	0.00
Calcitic limestone	0.89	0.90	0.62	0.44	0.00	0.00	0.00
Premix ^5^	0.20	0.20	0.20	0.20	0.20	0.20	0.20
BHT ^6^	0.02	0.02	0.02	0.02	0.02	0.02	0.02
Rice husk ^7^	0.00	0.00	0.00	0.00	0.00	7.77	0.00
Analyzed composition (%):							
Crude protein	25.76	31.99	31.87	28.85	36.93	38.85	45.16
Ether extract	9.54	14.13	10.51	10.52	13.91	16.34	10.29
Ash	10.45	10.39	7.36	9.97	10.62	11.65	8.93
Gross energy (kcal kg^−1^)	4260	4540	4062	4370	4650	4810	4540
Dry matter	92.94	93.38	92.64	93.14	93.60	93.78	92.72
Calculated composition (%):							
Digestible protein	22.5	25.0	27.5	30.0	32.5	35.0	37.5
Digestible energy (kcal kg^−1^)	3400	3400	3400	3400	3400	3400	3400
Starch	37.08	30.13	24.79	24.56	22.63	8.59	14.05
Fat	9.39	11.58	12.73	11.25	9.66	13.41	9.82
Neutral detergent fiber	7.76	7.52	7.26	7.09	6.21	3.7	4.86
Acid detergent fiber	2.28	2.51	2.64	2.56	2.19	1.55	1.9

^1^ Crude protein, 6.82%; ether extract, 2.94%; mineral matter, 0.97%; dry matter, 83.25%; gross energy, 3610 kcal/kg. ^2^ Crude protein, 48.93%; ether extract, 21.25%; mineral matter, 23.77%; dry matter, 95.40%; gross energy, 4680 kcal/kg. ^3^ Soybean protein concentrate: crude protein, 55.89%; ether extract, 0.22%; mineral matter, 5.99%; dry matter, 84.05%; gross energy, 3960 kcal/kg. ^4^ Crude protein, 62.72%; ether extract, 0.05%; mineral matter, 0.70%; dry matter, 62.87%; gross energy, 3520 kcal/kg. ^5^ Micromineral and Vitamin Supplement, Tectron Animal Nutrition and Health, Toledo, Paraná, Brazil. ^6^ Butylated hydroxytoluene, 12.25 g. ^7^ Crude protein, 2.65%; ether extract, 0.77%; mineral matter, 8.76%; dry matter, 9.74%; gross energy, 3750 kcal/kg.

**Table 2:** The effect of dietary digestible protein (DP) levels on the average cross-sectional area of cells and the number of muscle cells in white muscle.

DP Levels (g kg^−1^)	Class 1 (<11.4 μm^2^)	Class 2 (11.4–40.08 μm^2^)	Class 3 (40.08–230.18 μm^2^)	Class 4 (230.18–1133.73 μm^2^)	Class 5 (1133.73–11,777 μm^2^)
	Cross-Sectional Area of White Muscle Cells (μm^2^)				
225	6.49	33.71	188.34	967.29	9079.19
250	6.79	30.86	197.97	969.99	7935.07
275	7.26	33.11	193.13	1056.96	7159.36
300	6.84	31.36	191.17	968.77	7158.85
325	6.71	30.68	202.80	1039.74	6871.71
350	6.38	32.20	206.19	957.68	7576.19
375	6.90	31.77	185.66	1016.65	6812.05
SEM	0.832	1.73	9.38	46.27	839.34
Linear effect	0.968	0.491	0.679	0.623	0.055
Quadratic effect	0.728	0.446	0.278	0.510	0.202
	Number of white muscle cells				
225	107.89	113.12	126.11	115.52	113.00
250	150.70	107.45	106.96	107.99	125.41
275	173.45	162.93	146.03	120.2	137.67
300	137.48	154.54	150.97	145.75	111.73
325	95.99	110.37	130.51	145.2	129.26
350	100.67	100.22	97.33	114.62	115.07
375	83.81	112.83	104.52	113.31	129.67
SEM	51.58	28.07	23.25	18.38	11.24
Linear effect	0.304	0.604	0.362	0.712	0.690
Quadratic effect	0.338	0.179	0.142	0.120	0.703

SEM: standard error of the mean.

**Table 3:** The effect of digestible protein (DP) levels in the diet on the concentrations of total protein, free amino acids, and ammonia in the white muscle of Amazonian Pintado.

DP Levels (g kg^−1^)	Total Proteins (mg mL^−1^)	Free Amino Acids (μmol g^−1^ tissue)	Ammonia (μmol g^−1^ tissue)
225	1.5118	12.80	0.0410
250	1.7081	14.31	0.0426
275	1.6769	14.44	0.0367
300	1.6203	15.20	0.0388
325	1.8568	16.17	0.0358
350	1.7570	15.07	0.0395
375	2.1293	15.59	0.0372
SEM	0.1763	0.708	0.0029
Linear effect	0.0138	0.0028	0.2161
Quadratic effect	0.4488	0.1058	0.4758

SEM: standard error of the mean.

**Table 4:** The effect of dietary digestible protein (DP) levels on the concentrations of glycogen, glucose, lactate, and fat in the muscle of Amazonian Pintado.

DP Levels (g kg^−1^)	Glycogen (μmol g^−1^ tissue)	Glucose (μmol g^−1^ tissue)	Lactate (μmol g^−1^ tissue)	Fat (mg g^−1^ tissue)
225	8.98	0.3980	5.49	10.00
250	8.44	0.3407	6.08	8.403
275	7.25	0.3232	5.59	8.393
300	8.36	0.3945	6.11	9.164
325	7.39	0.3371	5.25	9.311
350	6.72	0.2648	5.25	10.950
375	7.32	0.313	6.66	9.572
SEM	0.782	0.038	0.71	1.258
Linear effect	0.0503	0.0574	0.6866	0.6511
Quadratic effect	0.5664	0.9903	0.5651	0.3406

SEM: standard error of the mean.

**Table 5:** The effect of dietary digestible protein (DP) levels on the concentrations of TBARS and carbonylated proteins in the muscle of Amazonian Pintado.

DP Levels (g kg^−1^)	TBARS (nmol MDA mg^−1^ protein)	Carbonylated Proteins (nmol Carbonyl mg^−1^ protein)
225	10.84	0.3808
250	6.54	0.3262
275	8.34	0.3905
300	11.59	0.3633
325	14.01	0.3911
350	13.31	0.4192
375	14.28	0.3943
SEM	2.94	0.05
Linear effect	0.0465	0.3965
Quadratic effect	0.6344	0.8674

SEM: standard error of the mean.

**Table 6:** The effect of dietary digestible protein (DP) levels on SOD, GSH, and GST concentrations in the muscle of Amazonian Pintado.

DP Levels (g kg^−1^)	SOD (IU SOD mg^−1^ protein)	GSH (μmol GSH mg^−1^ protein)	GST (μmol GS-DNB min^−1^ mg^−1^ protein)
225	9.85	0.0088	0.0865
250	9.87	0.0102	0.0826
275	10.31	0.0139	0.0803
300	10.22	0.0123	0.0900
325	9.09	0.0102	0.0832
350	9.06	0.0090	0.0449
375	9.67	0.0121	0.0495
SEM	0.631	0.002	0.009
Linear effect	0.3158	0.6899	0.0001
Quadratic effect	0.8000	0.3147	0.0311

SEM: standard error of the mean.

## Data Availability

Data are contained within this article.
